# Verbal intelligence is a more robust cross-sectional measure of cognitive reserve than level of education in healthy older adults

**DOI:** 10.1186/s13195-021-00870-z

**Published:** 2021-07-12

**Authors:** R. Boyle, S. P. Knight, C. De Looze, D. Carey, S. Scarlett, Y. Stern, I. H. Robertson, R. A. Kenny, R. Whelan

**Affiliations:** 1 Trinity College Institute of Neuroscience, Trinity College Dublin, Dublin, Ireland; 2 The Irish Longitudinal Study on Ageing, Trinity College Dublin, Dublin, Ireland; 3 Cognitive Neuroscience Division, Department of Neurology, Columbia University, New York City, USA; 4 Global Brain Health Institute, Trinity College Dublin, Dublin, Ireland; 5 Mercer’s Institute for Successful Ageing, St. James’s Hospital, Dublin, Ireland

**Keywords:** Cognitive reserve, Cognitive ageing, Cognitive decline, Neuroimaging, Structural MRI

## Abstract

**Background:**

Cognitive reserve is most commonly measured using socio-behavioural proxy variables. These variables are easy to collect, have a straightforward interpretation, and are widely associated with reduced risk of dementia and cognitive decline in epidemiological studies. However, the specific proxies vary across studies and have rarely been assessed in complete models of cognitive reserve (i.e. alongside both a measure of cognitive outcome and a measure of brain structure). Complete models can test independent associations between proxies and cognitive function in addition to the moderation effect of proxies on the brain-cognition relationship. Consequently, there is insufficient empirical evidence guiding the choice of proxy measures of cognitive reserve and poor comparability across studies.

**Method:**

In a cross-sectional study, we assessed the validity of 5 common proxies (education, occupational complexity, verbal intelligence, leisure activities, and exercise) and all possible combinations of these proxies in 2 separate community-dwelling older adult cohorts: The Irish Longitudinal Study on Ageing (TILDA; *N* = 313, mean age = 68.9 years, range = 54–88) and the Cognitive Reserve/Reference Ability Neural Network Study (CR/RANN; *N* = 234, mean age = 64.49 years, range = 50–80). Fifteen models were created with 3 brain structure variables (grey matter volume, hippocampal volume, and mean cortical thickness) and 5 cognitive variables (verbal fluency, processing speed, executive function, episodic memory, and global cognition).

**Results:**

No moderation effects were observed. There were robust positive associations with cognitive function, independent of brain structure, for 2 individual proxies (verbal intelligence and education) and 16 composites (i.e. combinations of proxies). Verbal intelligence was statistically significant in all models. Education was significant only in models with executive function as the cognitive outcome variable. Three robust composites were observed in more than two-thirds of brain-cognition models: the composites of (1) occupational complexity and verbal intelligence, (2) education and verbal intelligence, and (3) education, occupational complexity, and verbal intelligence. However, no composite had larger average effects nor was more robust than verbal intelligence alone.

**Conclusion:**

These results support the use of verbal intelligence as a proxy measure of CR in cross-sectional studies of cognitively healthy older adults.

**Supplementary Information:**

The online version contains supplementary material available at 10.1186/s13195-021-00870-z.

## Background

Neuropathology and measures of brain structure do not fully explain cognitive decline [1] nor age-related variation in cognitive function [2]. This is evident in the finding of normal cognitive function in individuals who meet the diagnostic criteria for Alzheimer’s disease (AD) based on neuropathology [3, 4]. This well-established gap between brain and cognition may be explained by *cognitive reserve* (CR), wherein the effects of brain pathology or ageing on cognitive function are moderated by an individual’s ability to efficiently or flexibly use the brain’s resources to cope with task demands [5].

Accurate measurement of CR could improve the detection of, and risk assessments for, age-related cognitive decline and AD [6] and improve the measurement of intervention efficacy in clinical trials and intervention studies by enabling researchers to effectively statistically control for CR [7]. Difficulties in measuring CR [8], however, limit this potential. The most direct measures of CR are likely to be obtained using functional neuroimaging [8]. CR may be measured with functional MRI using resting-state and task-based functional connectivity. For example, a pattern of greater change in functional connectivity from resting-state in response to task demands is associated with better cognitive performance, above and beyond the effects of cortical thickness [9]. However, the considerable cost of MRI scanning [10] limits access to such measures, particularly in lower income countries [11]. As such, socio-behavioural variables reflecting the degree of exposure to, or engagement in, various lifetime experiences are often used as proxies of CR [8].

The rationale for using proxies is that greater exposure to certain lifetime experiences increases the adaptability of cognitive and functional brain processes, thereby enabling a greater ability to cope with brain changes or damage [8]. Considerable epidemiological evidence indicates a reduced risk and/or delayed onset of dementia and cognitive decline in individuals with greater educational attainment [12–14], occupational complexity/status [15–17], literacy and/or verbal intelligence [18–21], engagement in activities that were cognitively stimulating [22, 23], leisure-related [24, 25], physical [22, 26–28], and social [22, 23, 29]. Proxies also provide a single value with a simple interpretation—a higher degree of exposure reflects greater CR. Moreover, proxies are easy and inexpensive to obtain, and some, such as educational attainment, are routinely collected as part of most ageing studies. It is therefore not surprising that CR is most often measured using proxies [30].

Despite their advantages, the use of proxies to measure CR has been criticized. First, some proxies, such as educational attainment, are typically static measures [31] despite the fact that CR is considered to be a dynamic construct that can change over time [32]. Second, some argue that a single proxy fails to reflect the full CR construct which is thought to be influenced by a range of experiences [33, 34]. Finally, proxies may also be associated with cognitive decline via mechanisms other than reserve [35]. For instance, greater educational attainment is correlated with higher socioeconomic status [36] which is itself associated with slower cognitive decline [37] and reduced risk and prevalence of dementia [38, 39]. Low socioeconomic status is associated with various other factors, including stress and access to healthcare, which could exacerbate cognitive decline [38]. As such, the protective effect of education on cognitive decline and dementia (but cf. [40] for an alternative perspective) may be via mechanisms related to socioeconomic status, rather than CR [41].

The limitations of individual proxies may be mitigated by averaging (cf. transformation methods such as principal component analysis) multiple proxies to create a composite proxy measure that still provides a single summary value with a simple interpretation [42–46]. Composite proxies allow for a wider range of contributions to CR and enable the inclusion of dynamic proxies that can change over time, such as verbal intelligence or engagement in activities [31]. Furthermore, composite proxies may attenuate the issue of non-CR mechanisms of individual proxies because alternative mechanisms (e.g. socioeconomic status) might only be associated with some proxies, such as educational attainment, but not others like social engagement. Some composite-type approaches, including factor analytic and latent variable models, measure CR using inappropriate *reflective measurement models*, where the observed CR proxies are effectively considered to be reflective (i.e. caused by) the latent CR construct [35]. Composite proxies are a more appropriate formative measurement model, where the observed proxies are considered to form, or cause, CR. Moreover, this approach can reflect the unique additive contributions of individual proxies, whereas factor analytic models reflect only the shared variance across different proxies [8].

While the composite approach offers advantages over the use of single proxies, there is no agreed-upon gold-standard composite proxy [30] just as there is likewise no gold-standard individual proxy. Similarly, it is unclear which proxy should be used when assessing candidate neuroimaging measures of CR, as face validity is assessed via their association with CR proxies [47, 48]. The considerable variation [49, 50] and lack of coherence in the use of proxies means that there is poor comparability across studies, as an effect observed for one proxy (e.g. educational attainment) may not be observed to the same degree for another (e.g. occupational complexity), even though both putatively reflect CR. It also provides researchers in the field of CR with an additional “researcher degrees of freedom” [51] such that several different proxies could be examined but only statistically significant results are reported.

To assess the validity of a potential measure of CR, a complete model of CR is required, which includes 3 components: a measure of CR (e.g. a proxy), a measure of brain structure/pathology, and a measure of cognitive function [8, 52]. This enables the assessment of the *cognitive benefit criterion* [48]. This criterion can be satisfied via the observation of (1) an “independent effect” in which the candidate measure is positively associated with cognitive function, independent of brain structure, or (2) a “moderation effect” in which the candidate measure moderates the relationship between brain structure and cognitive function [8, 47]. The moderation effect is considered the ideal benchmark for CR, whereas the independent effect is considered a weaker level of evidence for a CR effect [8].

A systematic review of CR proxies from complete CR models reported inconclusive evidence for educational attainment, occupational complexity/status, and leisure activity as proxies of CR in cognitively healthy cohorts [53]. A single reviewed study provided evidence that greater engagement in cognitively stimulating activities in mid- and late-life provided CR effects [54]. Other proxies were not assessed in this systematic review, although individual studies have reported positive evidence for CR effects in complete CR models. Verbal intelligence has been positively associated with cognition, controlling for global AD neuropathology or hippocampal atrophy in cognitively healthy [55, 56] and cognitively impaired older adults [55]. Physical activity was positively associated with cognition in the presence of neuropathology [57] but not hippocampal atrophy [56]. Social engagement moderated the relationship between amyloid-beta deposition and cognitive decline [58]. The composite of verbal intelligence and education moderated the relationship of subcortical grey matter (GM) volume and cortical thickness with fluid reasoning but not memory or processing speed and attention [46]. This composite was also associated with memory controlling for GM volume [59] and global cognition controlling for a composite AD-biomarker [45]. Although other composites have been associated with cognition [50], there is very little empirical evidence regarding their effects within complete CR models.

There is currently no conclusive evidence for the best individual or composite proxy for measuring or validating neuroimaging measures of CR, particularly with respect to cognitively healthy older adults. A methodology for solving this problem is the use of hierarchical linear moderated regressions to systematically assess standard CR proxies and their composites in complete models, an approach that enables the examination of both moderation and independent effects within the same analysis framework. This is important because, although moderation effects should ideally be observed to validate a CR proxy or measure [8], they are typically small in real-world data [60], explaining 1–3% of the variance in the outcome [61]. Consequently, large sample sizes are required to detect typically small moderation effects [62]. This issue is further exacerbated when measurement error is present in either variable in the interaction term (e.g. the CR proxy and measure of brain structure) used to assess the moderation effect [63] or when either variable in the interaction term is associated with the outcome variable (e.g. cognitive function [64];). Given the noted difficulties in identifying moderation effects, it is important to also consider the independent effect when assessing the validity of CR proxies.

Hierarchical linear regressions allow the robustness (i.e. frequency of effects using different measures of brain structure and cognitive function) and magnitude of both moderation and independent effects of different proxies to be compared. Here, in two separate community-dwelling older adult cohorts, we examined five common putative CR proxies—education, occupational complexity, verbal intelligence, leisure activities, and exercise—and all of their possible combinations. We included three brain structure variables, mean cortical thickness, hippocampal volume, and grey matter volume, in each model. Our primary aim was to identify the CR proxies with the most robust and largest effects across two datasets. More formally, we define effective CR proxies as those variables that have a significant independent or moderation effect on measures of cognitive function and brain structure.

## Method

### Participants

The first dataset consisted of data from 313 community-dwelling adults (mean age = 68.90 years, SD = 6.75 years, range = 54–88 years; 50.48% female), a subset of The Irish Longitudinal Study on Ageing (TILDA), and a nationally representative longitudinal cohort study of older adults in Ireland [64, 65]. This data was collected during Wave 3 of the TILDA study [66]. All participants were screened for MRI contraindications, and study-specific inclusion criteria included no history of neurological conditions and available data for CR proxies and cognitive function.

The second dataset consisted of data from 234 community-dwelling adults (mean age = 64.49 years, SD = 7.42 years, range = 50–80 years; 51.28% female) selected from participants in the Cognitive Reserve/Reference Ability Neural Network (CR/RANN) studies [67–69]. Participants were screened for MRI contraindications, hearing and visual impairments, medical or psychiatric conditions, and dementia or MCI. Participants selected for the current analyses were aged 50 years or older with data available for CR proxies, cognitive function, and MRI.

### Measures: CR proxies

Data was available for 5 socio-behavioural proxies in both datasets: *educational attainment*, *occupational complexity*, *verbal intelligence*, *leisure activities*, and *physical activity*. In TILDA, further data was available for the proxies: *cognitively stimulating activities* and *social engagement*.

*Educational attainment* was measured using years of formal education in both datasets. In TILDA, participants were asked to indicate the age at which they first left continuous full-time education. This information was missing for 4 participants in the final sample (1.28%), so it was imputed using educational qualification, father’s education, age, sex, and rural residence during childhood as previously described [70].

*Occupational complexity* was measured using the complexity of work in the dimensions of data, people, and things [71] using ratings obtained from an online catalogue of the Dictionary of Occupational Tiles (DOT: www.occupationalinfo.org). Ratings for each dimension were reversed (such that higher scores reflected greater complexity) and then summed to create a total occupational complexity score, with scores ranging from 0 (minimal complexity) to 21 (maximal complexity). This was obtained for each participant’s current occupation or last occupation before retirement in TILDA and for participant’s occupation of longest duration of their lifetime in CR/RANN.

*Verbal intelligence* was measured using the total number of correctly pronounced words on the National Adult Reading Test (NART; Nelson and Willinson [72]) in TILDA and the American National Adult Reading Test (AMNART; Grober and Sliwinski [73]) in CR/RANN. In TILDA, a stress/anxiety-preventative and time-saving measure [74] was employed such that participants only completed the second half of the NART if they scored greater than 20 on the first half. A correction procedure was employed whereby scores of 0–11 were retained as full scores, but scores of 12–20 in participants who did not complete the second half were corrected using a conversion table outlined by Beardsall and Brayne [75, 76]. Possible scores on the NART, in TILDA, ranged from 0 to 50 and on the AMNART, in CR/RANN, from 0 to 45. While the NART is often used to provide a measure of premorbid intelligence, we have labelled NART scores here as verbal intelligence in line with previous cognitive reserve studies [42, 77]. The NART is “effectively a test of knowledge acquisition” [78] that may reflect the exposure to various educational and cognitive experiences across the lifespan [79–82].

*Leisure activities* were assessed in TILDA by participants rating their current frequency of engagement on an 8-point Likert scale (0 = never to 7 = daily/almost daily) in 9 activities: *watching television*, *going to films/plays/concerts*, *travel*, *listening to music/radio*, *going to the pub*, *eating out*, *sports/exercise*, *visiting/talking on phone*, and *volunteering*. In CR/RANN, participants rated their frequency of engagement over the preceding 6 months on a 3-point Likert scale (1 = never to 3 = often) in 17 activities: *television/radio*, *cards/games*, *reading*, *lectures/concerts*, *theatre/movies*, *travel*, *walks/rides*, *crafts/hobbies*, *music*, *visiting*, *sports/dancing/exercise*, *cooking*, *group membership*, *collecting*, *religious activities*, and *volunteering*. For both datasets, total scores were created by summing individual responses and possible scores ranged from 17 to 51.

*Physical activity* was assessed in TILDA by calculating the total metabolic minutes arising from self-reported physical activity over the last week using the International Physical Activity Questionnaire-Short Form (IPAQ-SF; Craig et al. [83]; Lee et al. [84]). This questionnaire assessed the time spent in 3 categories: vigorous, moderate, and walking. Responses were converted to metabolic equivalent minutes [83] and summed. In CR/RANN, physical activity was calculated using total metabolic hours arising from physical activity in an average week. The Godin leisure-time exercise questionnaire [85] assessed the frequency of activity sessions lasting > 15 min in 3 categories: strenuous, moderate, and mild exercise. Responses were then weighted by the average estimated duration of activity in each category (0.5, 0.75, and 1 h, respectively) and their metabolic equivalent values (9, 5, 3; Ogino et al., [28]; Scarmeas et al. [86]).

*Cognitively stimulating activities* were assessed in TILDA with a questionnaire where participants rated their frequency of engagement on an 8-point Likert scale (0 = never to 7 = daily/almost daily) in 5 activities: *attending classes and lectures*, *working in the garden/home or on a car*, *reading books/magazines*, *spending time on hobbies/creative activities*, and *playing cards/bingo/games*. Total scores were created by summing individual responses and possible scores ranged from 0 to 35.

*Social engagement* was measured in TILDA using the Social Network Index [87] which provides a total score, ranging from 0 to 4, reflecting an individual’s degree of social connection [88].

*Composite proxies* were created by first standardizing (z-scoring) individual proxies. Next, every unique combination of proxies was generated and the composite proxy was the average of those proxies. For TILDA, this produced 120 unique composite proxies. For CR/RANN, this resulted in 26 composite proxies.

To summarize, for TILDA, there were 127 proxies in total (individual and composite) and 31 in total for CR/RANN. To attenuate possible effects of outliers, all proxies were Winsorized using a robust technique based on the median absolute deviation [89]. Outliers were identified as values greater than a threshold of 3 median absolute deviations from the median. Identified outliers were replaced by the median ± 3 median absolute deviations.

### Measures: cognitive function

*Verbal fluency* was assessed using the total score on the Animal Naming Test which measures the ability to spontaneously produce the name of animals in 1 min [74]. The total number of animals named was used as the total score in both datasets.

*Processing speed* was measured using the time to complete the Colour Trails Task 1 (CTT 1; D’Elia et al. [90]) in TILDA and the Trail Making Task A (TMT A; Reitan [91]) in CR/RANN. The CTT is considered a cross-culturally valid form of the TMT [74]. Scores were reversed coded, such that higher scores reflected greater cognitive performance.

*Executive function* was assessed using the CTT 2 (D’Elia et al. [90]) in TILDA and the TMT B (Reitan [91]) in CR/RANN. Both measures reflect the multi-dimensional executive function construct [92, 93], specifically visual attention and cognitive flexibility with contributions from processing speed as well [74]. The time taken to complete both tasks was used as the outcome measure. Scores were reverse coded such that higher scores reflected greater cognitive performance.

*Episodic memory* was measured in both datasets with a composite measure created using the average of standardized and Winsorized immediate and delayed recall variables. In TILDA, immediate and delayed recall were measured using a 10-item word list [94] as used originally in the Health and Retirement Study [95]. The word list was assessed over 2 trials in TILDA and the average score for immediate and delayed recall from both trials was used. In CR/RANN, immediate and delayed recall were measured using the total and delayed recall scores from the Selective Reminding Test (SRT; Buschke and Fuld [96]).

*Global cognition* was measured using a composite measure of all 5 cognitive variables in each dataset: verbal fluency, processing speed, executive function, episodic memory (immediate recall), and episodic memory (delayed recall). Cognitive variables were Winsorized and standardized prior to creation of the composite. The composite variable was then Winsorized and standardized itself.

### Measures: brain structure

T1-weighted 3D magnetization-prepared rapid gradient echo (MPRAGE) scans were acquired in both datasets using a 3-T scanner (Achieva, Philips Medical Systems, The Netherlands). TILDA parameters: FOV = 240 × 240 × 162 mm^3^, matrix size = 288 × 288, slice thickness/gap = 0.9/0 mm, TR/TE = 6.7/3.1 ms. CR/RANN parameters: FOV = 256 × 256 × 180 mm^3^, matrix size = 256 × 256, slice thickness/gap = 1/0 mm, TR/TE = 6.5/3 ms.

T1-MRIs were inspected and processed in TILDA and CR/RANN using FreeSurfer v6.0 and v5.1 [97], respectively, as described previously [68, 98]. Total GM volume and hippocampal volume were obtained from Freesurfer and both were divided by Freesurfer’s estimated total intracranial volume. Brain images were parcellated using the Desikan Killiany atlas, with 34 cortical regions of interest (ROIs) per hemisphere [99]. The mean cortical thickness of each cortical ROI was calculated. Overall cortical thickness was calculated as the mean over cortical ROIs. All variables were standardized and Winsorized (based on z-scores > |3|). These three measures were selected based on their availability across both datasets and because they have been used in previous studies, with complete CR models, to represent brain structure: GM volume [100, 101], hippocampal volume [102, 103], and mean cortical thickness [9, 43, 104].

### Analysis

**Fig. 1:**
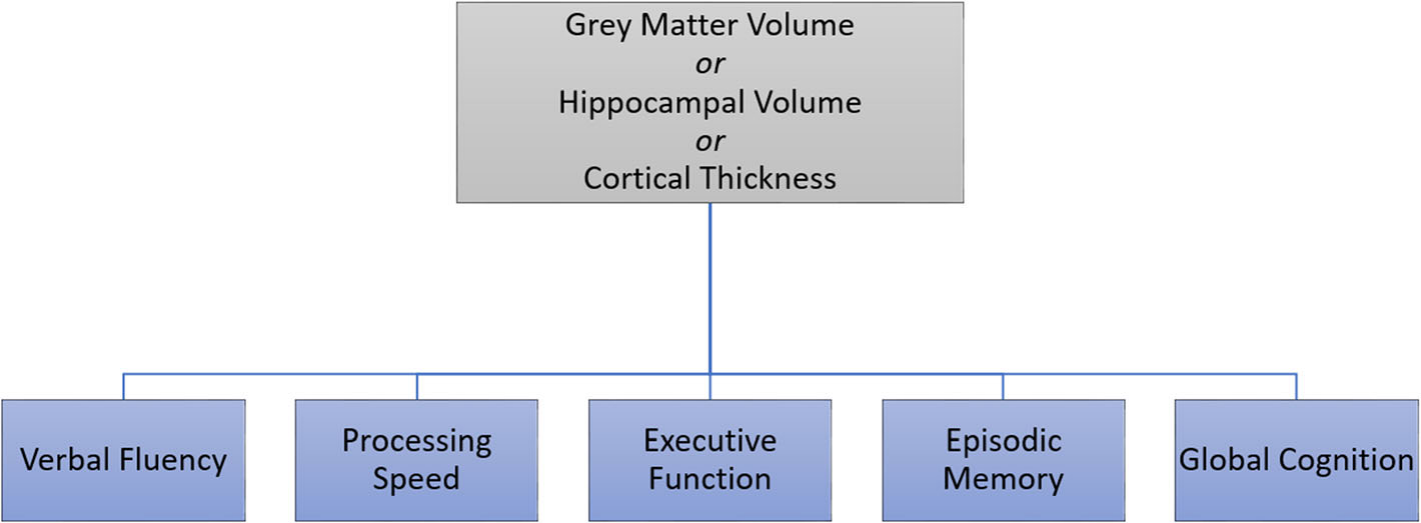
Schematic of basic brain structure-cognitive function models created for analysis

**Fig. 2:**
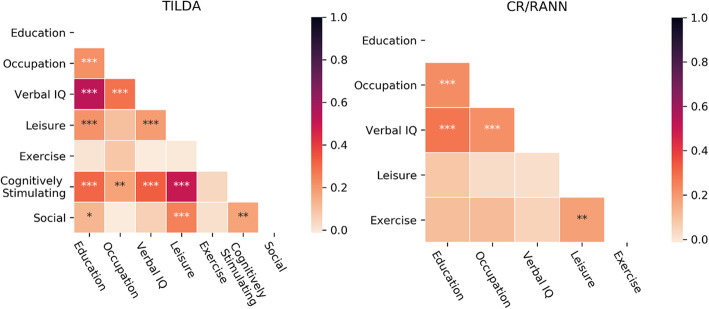
Heatmaps showing Pearson’s correlations between individual proxies in each dataset. *p < .05, **p < .01, ***p < .001

Fifteen individual brain structure-cognitive function models were created for each combination of brain structure and cognitive function variable, where one brain structure variable was selected as an independent variable and one cognitive function variable was selected as an outcome variable (Fig. 1). A moderated hierarchical regression (Fig. 1) was conducted within each brain structure-cognitive function model (*n* = 15) for each unique proxy (TILDA = 127; CR/RANN = 31). In step 1, a cognitive measure was regressed on age, sex, and a measure of brain structure. In step 2, a proxy variable (Fig. 2) was included as an independent variable. In step 3, the interaction term for brain structure and the proxy was added.

To protect against violations of linear regression assumptions, the analysis was repeated using a robust regression, specifically an iteratively reweighted least squares regression with Tukey’s biweight function and median absolute deviation scaling. Effects within each dataset were only considered significant if they were statistically significant in both the linear regression and robust regression. To control for multiple comparisons and to ensure generalizability of findings, effects were only considered significant if they were statistically significant across both datasets. The analysis was conducted with customized Python code (available here: https://github.com/rorytboyle/hierarchical_regression) which used the *statsmodels* module [105]. The change in R^2^ (i.e. amount of variance explained) from step 1 to step 2 and from step 2 to step 3 in linear regression models was used to assess the size of the independent and moderation effects of CR proxies, respectively. Where significant effects were observed, the mean R^2^ change across both datasets was calculated to assess the average additional variance explained by the proxy and its interaction with brain structure.

## Results

### Demographics

In TILDA, some data were missing for mean cortical thickness (*N* = 34) and CTT 2 and Global Cognition (*N* = 2). In CR/RANN, the same N was used (*N* = 234) in all models. Consequently, different Ns were used across models within TILDA (see Table 1).

**Table 1:** Demographics for each hierarchical regression model

Dataset	Brain structure	Cognition	N	Mean age (SD, range)	Sex (M/F)
TILDA	Grey matter volume, hippocampal volume	Verb Flu, Proc Speed, Epi Mem	313	68.90 (6.75, 54–88)	155/158
	Grey matter volume, hippocampal volume	Exec Func, Glob Cog	311	68.91 (6.77, 54–88)	154/157
	Mean cortical thickness	Verb Flu, Proc Speed, Epi Mem	279	69.16 (6.64, 54–88)	137/142
	Mean cortical thickness	Exec Func, Glob Cog	277	69.18 (6.66, 54–88)	136/141
CR/RANN	All	All	234	64.49 (7.42, 50–80)	114/120

*SD* standard deviation, *M* male, *F* female, *Verb Flu* verbal fluency, *Proc Speed* processing speed, *Epi Mem* episodic memory, *Exec Func* executive function, *Glob Cog* global cognition

### Step 1: Brain-cognition relationships

Models in step 1 of the hierarchical regression (i.e. containing a brain structure measure, sex, and age) were significantly associated with cognitive measures across both datasets (see Tables 2 and 3), except for two models in CR/RANN (hippocampal volume-executive function, and hippocampal volume-episodic memory). Sex was independently associated with cognitive function in 40% and 20% of brain-cognition models in TILDA and CR/RANN, respectively. In TILDA, females had higher cognitive function than males, on average, with other variables (i.e. brain structure and age) being equal. In CR/RANN, females had lower cognitive function than males, on average, with other variables being equal. Age was negatively associated with cognitive function, independent of brain structure and sex, in 100% and 40% of models in TILDA and CR/RANN, respectively.

**Table 2:** Step 1 of hierarchical regression models in TILDA

Cognition	Model statistics			Brain structure		Sex	Age
	***n***	***R***^***2***^	***f***	Variable	***β***	***β***	***β***
Verb Flu	313	.043	4.597**	Grey matter volume	.042	−.030	−.205**
Proc Speed	313	.129	15.320****		.041	.084	−.360****
Exec Func	311	.143	17.070****		.048	.052	−.383****
Epi Mem	313	.079	8.780****		.021	.352**	−.207**
Glob Cog	311	.159	19.400****		.048	.217*	−.373****
Verb Flu	313	.042	4.475**	Hippocampal volume	−.005	−.004	−.229**
Proc Speed	313	.129	15.226****		−.025	.120	−.394****
Exec Func	311	.143	17.010****		−.041	.101	−.428****
Epi Mem	313	.080	8.902****		.044	.341**	−.195**
Glob Cog	311	.158	19.171****		.002	.243*	−.396****
Verb Flu	279	.051	4.898**	Mean cortical thickness	.103	.002	−.192**
Proc Speed	279	.173	19.217****		.122*	.042	−.370****
Exec Func	277	.195	22.040****		.090	.065	−.428****
Epi Mem	279	.091	9.202****		−.036	.414**	−.216***
Glob Cog	277	.195	22.105****		.065	.251*	−.391****

*Verb Flu* verbal fluency, *Proc Speed* processing speed, *Exec Func* executive function, *Epi Mem* episodic memory, *Glob Cog* global cognition

*p < .05, **p < .01, ***p < .001, ****p < .0001

**Table 3:** Step 1 of hierarchical regression models in CR/RANN

Cognition	Model statistics			Brain		Sex	Age
	***n***	***R***^***2***^	***f***	Variable	***β***	***β***	***β***
Verb Flu	234	.087	7.320***	Grey matter volume	.258***	−.073	−.062
Proc Speed	234	.087	7.344***		.218**	−.296*	−.120
Exec Func	234	.047	3.762*		.175*	−.247*	−.063
Epi Mem	234	.061	4.998**		.221**	.070	−.072
Glob Cog	234	.130	11.498****		.330****	−.148	−.117*
Verb Flu	234	.043	3.449*	Hippocampal volume	.078	<−.001	−.111*
Proc Speed	234	.061	5.014**		.034	−.225	−.173**
Exec Func	234	.030	2.339		.026	−.190	−.107
Epi Mem	234	.033	2.608		.032	.142	−.127*
Glob Cog	234	.069	5.671***		.061	−.044	−.195**
Verb Flu	234	.065	5.303**	Mean cortical thickness	.166**	−.024	−.098
Proc Speed	234	.073	6.063***		.129	−.252*	−.152*
Exec Func	234	.048	3.834*		.153*	−.226	−.077
Epi Mem	234	.053	4.281**		.159*	.106	−.098
Glob Cog	234	.109	9.401****		.231***	−.092	−.158**

*Verb Flu* verbal fluency, *Proc Speed* processing speed, *Exec Func* executive function, *Epi Mem* episodic memory, *Glob Cog* global cognition

*p < .05, **p < .01, ***p < .001, ****p < .0001

In TILDA, only one brain structure variable, mean cortical thickness, was independently positively associated with cognitive function (processing speed). In CR/RANN, grey matter volume was independently positively associated with all cognitive measures and cortical thickness was independently positively associated with all cognitive measures except for processing speed. Hippocampal volume was not independently associated with any measure of cognition in either dataset.

### Step 2a: Independent effects

**Fig. 3:**
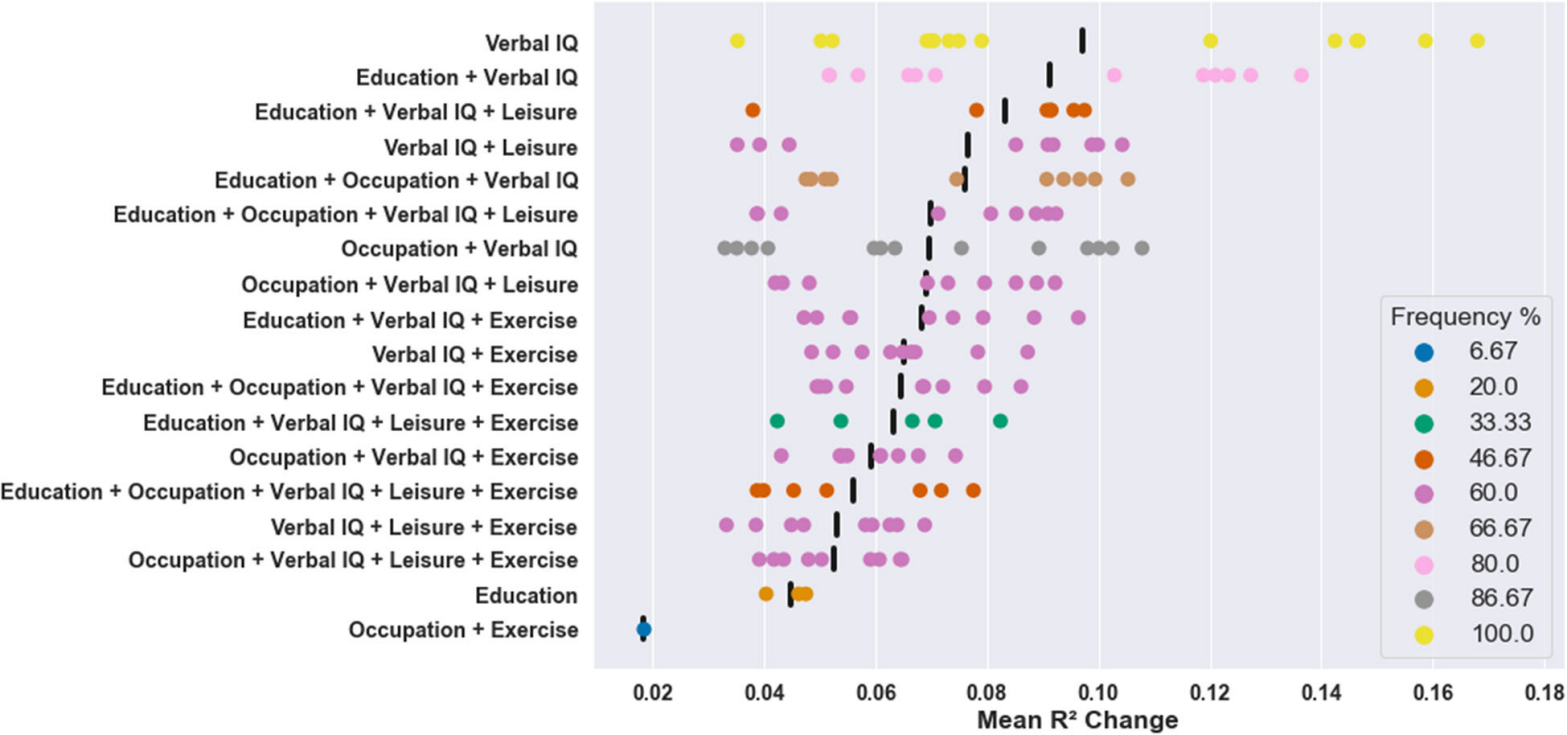
Mean R^2^ change across datasets in all models for proxies with significant effects. + indicates composite proxies (e.g. Education + Verbal IQ = composite of educational attainment and verbal intelligence). Black vertical bars represent the mean of significant R^2^ change values across all models for that proxy. All models were adjusted for brain structure, age, and sex

Significant positive independent effects were observed for 18 proxies, including 2 individual proxies and 16 composites, across the 15 models in both datasets (see Additional file 1 for significant independent effects across both datasets; see Additional file 2 for all significant independent effects in TILDA; see Additional file 3 for all significant independent effects in CR/RANN). The proxy with the largest average independent effect was verbal intelligence (mean R^2^ change = 0.10; see Fig. 3). Verbal intelligence was the most robust proxy: independent effects were replicated across both datasets in 100% of models. The largest average independent effects were observed for verbal intelligence on global cognition where it explained a mean additional 16.80% (hippocampal volume), 15.87% (grey matter volume), and 14.66% (mean cortical thickness) of the variance after accounting for age, sex, and brain structure (for scatter plots of proxies with 10 largest average independent effects, see Additional file 4, Fig. S1). Education was the only other individual proxy with reproducible independent effects (mean R^2^ change = 0.05), which were observed in 20% of models, all of which contained executive function.

The most robust composite proxy was comprised of occupational complexity and verbal intelligence (mean R^2^ change = 0.07) which was replicated in 86.67% of models. The composite proxy with the largest average effect was educational attainment and verbal intelligence (mean R^2^ change = 0.09) which was replicated in 80% of models. Only one composite with reproducible independent effects—occupational complexity and physical activity—did *not* include verbal intelligence. This was the least robust composite as it was replicated in a single model and had the smallest average effect (mean R^2^ change = 0.02).

### Step 2b: Additional independent effects

**Fig. 4:**
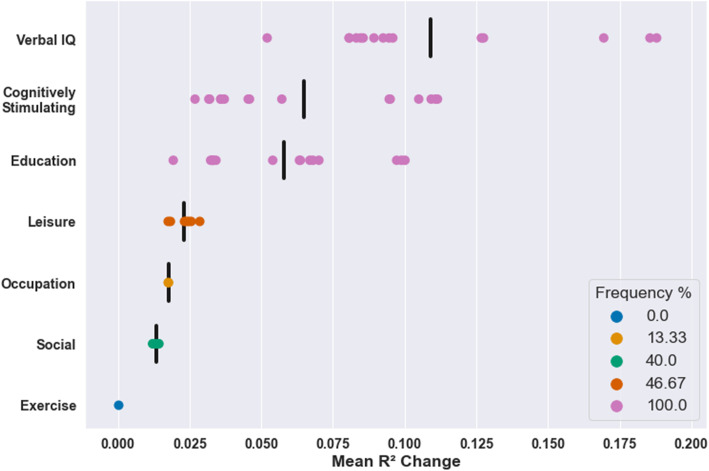
Mean R^2^ change of significant effects in all TILDA models for individual proxies. Black vertical bars represent the mean of significant R^2^ change values across all models for that proxy. All models were adjusted for brain structure, age, and sex

Data was only available for cognitively stimulating activities and social engagement in TILDA. Consequently, these effects could not be assessed in terms of their reproducibility. However, within TILDA, positive independent effects of cognitively stimulating activities on cognition were observed in 100% of models and this proxy had the second largest average independent effect of all individual proxies (mean *R*^2^ change = 0.065, see Fig. 4). In contrast, positive independent effects of social activities on cognition were observed in only 40% of models and this proxy had the second smallest average independent effect of all individual proxies (mean R^2^ change = 0.013). The only individual proxy with smaller effects than social engagement was the physical activity which did not have significant effects in any model.

**Fig. 5:**
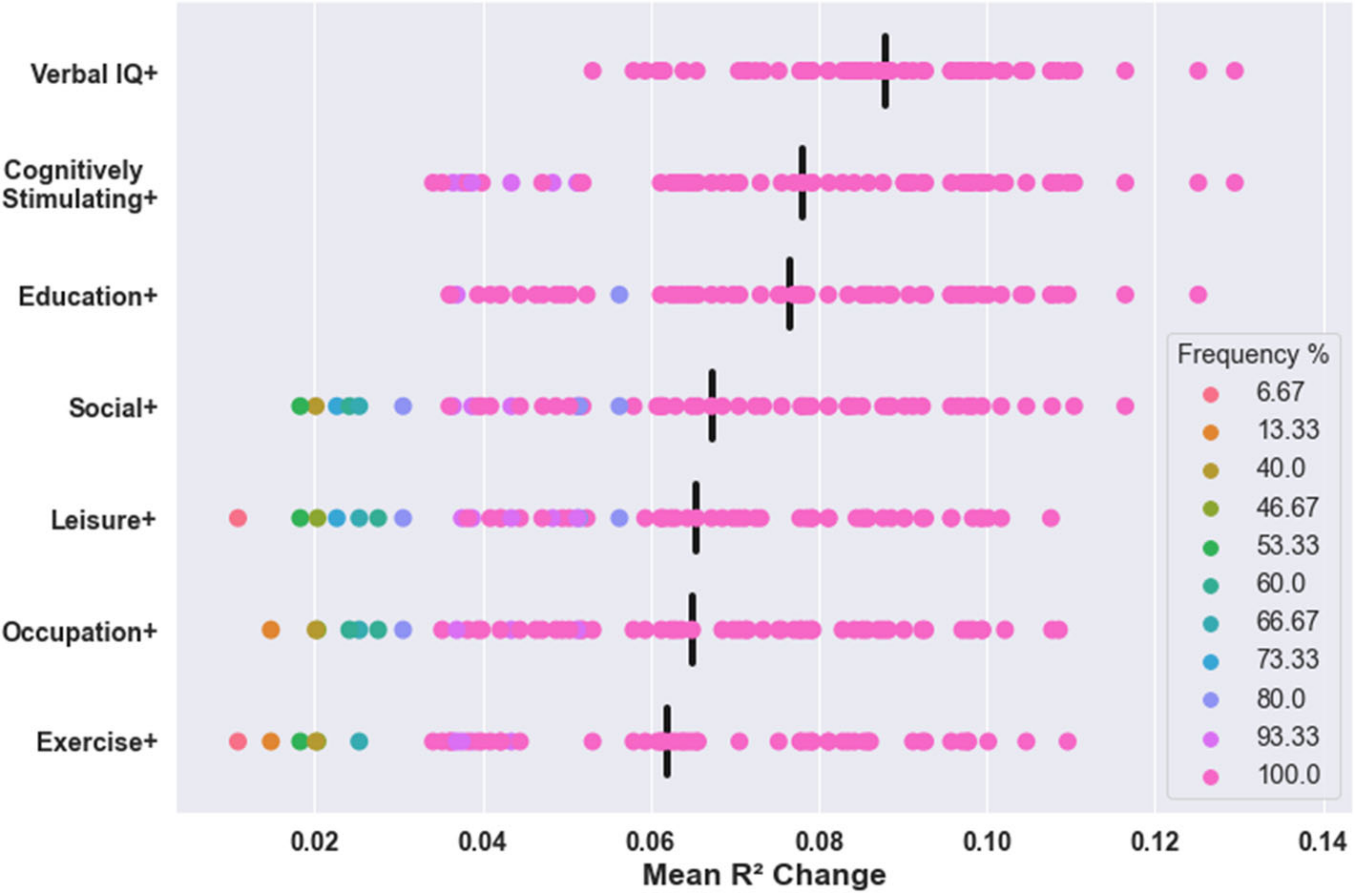
Mean R^2^ change of significant effects in all TILDA models for composite proxies. Each row refers to all composites including that proxy (e.g. Verbal IQ+ refers to all composites including verbal intelligence). Black vertical bars represent the mean of significant R^2^ change values across all models for all composites containing that proxy. All models were adjusted for brain structure, age, and sex

Composite proxies including verbal intelligence had the largest average effects, followed by cognitively stimulating activities, and then education (see Fig. 5). Composites including verbal intelligence had significant effects in all models in TILDA. The composite with the largest effect in TILDA was verbal intelligence and cognitively stimulating activities (mean R^2^ change = 0.13). The only composite proxy which was not significant in any model was social engagement and physical activity.

### Step 3: Moderation effects

There were no significant moderation effects, in either dataset for any proxy, on the association between brain structure—as measured by GM volume, hippocampal volume, or mean cortical thickness—and cognition. Negative moderation effects are consistent with the CR hypothesis because they reflect weaker associations between brain structure and cognition in individuals with higher CR, suggesting that individuals with higher CR are less reliant on brain structure to sustain cognitive function. Thirty-one non-replicated negative moderation effects (i.e. consistent with the CR hypothesis) were observed in TILDA (see Additional file 4, Table S1), but none survived correction for multiple comparisons (Bonferroni-adjusted alpha = 0.0004: alpha [0.05]/comparisons per model [106]). 61.29% of these effects were observed for composite proxies including cognitively stimulating activities, which was not available in CR/RANN. No negative moderation effects were observed in CR/RANN.

Positive moderation effects contradict the CR hypothesis as they reflect stronger associations between brain structure and cognition in individuals with higher CR, suggesting that individuals with higher CR are more reliant on brain structure to sustain cognitive function. Non-replicated positive moderation effects (i.e. contradicting the CR hypothesis) were observed in both datasets (see Additional file 4, Table S2), but none survived correction for multiple comparisons. Eight effects were observed in TILDA (Bonferroni-adjusted alpha = 0.0004) and seven effects were observed in CR/RANN (Bonferroni-adjusted alpha = 0.0016: alpha [0.05]/comparisons per model [31]). The Bonferroni corrections for multiple comparisons applied here are liberal as they correct for the number of proxies compared per brain-cognition model (TILDA 127, CR/RANN 31) rather than the number of total comparisons across all proxies and all brain-cognition models (TILDA 1905; CR/RANN 465).

## Discussion

The reproducibility and magnitude of moderation and independent effects of 33 CR proxies, comprised of 5 standard individual proxies and all their unique combinations, were assessed across 2 datasets to investigate their validity as measures of CR. No moderation effects of CR proxies on the association between brain structure—as measured by GM volume, hippocampal volume, or mean cortical thickness—and cognition were observed across both datasets. Replicated independent effects—positive associations with cognitive function, independent of brain structure—were observed for 2 individual proxies (verbal intelligence and educational attainment) and 16 composites. The most robust and largest effects on cognition were found for verbal intelligence, which satisfied the independent effect criterion in all 15 brain-cognition models across both datasets. Educational attainment satisfied the independent effect criterion in 3 brain-cognition models. No composite proxy had larger or more robust independent effects on cognition than verbal intelligence alone. Our results support the use of verbal intelligence as a proxy measure of CR in cross-sectional studies of cognitively healthy older adults.

### Verbal intelligence had larger and more robust effects on cognition than educational attainment

We found that verbal intelligence had the largest and most robust independent effects on cognition. Unlike previous studies, due to the availability of two large neuroimaging datasets, we could demonstrate that independent effects of verbal intelligence on cognition were present in several brain-cognition models and were replicable. This validation of verbal intelligence as a CR proxy supports previous, narrower, associations between verbal intelligence and cognitive function in the presence of hippocampal atrophy [56], a neuropathological ‘residual’ measure of CR [55], a functional connectivity measure of CR based on task potency [9], and a possible neuromarker of CR, locus coeruleus signal intensity [107].

Aside from verbal intelligence, the only other individual proxy with replicable independent effects on cognition was educational attainment. These replicable effects were only observed in brain-cognition models where executive function was the cognitive outcome variable. While education has been previously positively associated with executive function, without accounting for brain structure, in cognitively healthy older adults [108] and in a systematic review [50], our results show that this association is independent of GM volume, hippocampal volume, or mean cortical thickness. Notably, the effects of education were less robust than verbal intelligence, as positive associations were not seen across both datasets for verbal fluency, processing speed, episodic memory, and global cognition. As such, these results suggest that educational attainment is not a reliable individual proxy of CR in cognitively healthy older adults. This conclusion is supported by previous findings including a systematic review which found positive evidence for education in only 38% of complete models with cognitively healthy samples [53] and a non-significant association between education (when considered separately from other possible CR proxies) and a neuropathological residual measure of CR [54]. Based on their findings using ex-vivo neuropathological measures, Reed et al. [54] concluded that the observed effects of education on cognition should not be simply considered as reserve effects. Our results further show that this conclusion is valid when using in-vivo neuroimaging measures of GM volume, hippocampal volume, or mean cortical thickness.

The general finding that verbal intelligence had larger and more robust CR effects than educational attainment convincingly supports an argument favouring the use of verbal intelligence over education [79]. This argument was previously broadly supported by evidence that, compared to educational attainment, verbal intelligence was a stronger predictor of cognitive function/decline [109, 110] and had greater protective effects on the onset of clinical symptoms of MCI/AD [43, 111]. More specifically, Malek-Ahmadi et al. [31] directly compared educational attainment and verbal intelligence in a mixed autopsy sample, consisting of adults with diagnoses of no cognitive impairment, MCI, and AD. In complete CR models, including neuropathological indices and measures of episodic memory and executive function, positive evidence was found for verbal intelligence, but not education, as a CR proxy, leading to the conclusion that verbal intelligence measures are superior to educational attainment as CR proxies. Here, we have shown that verbal intelligence is also a superior CR proxy when using in-vivo measures of GM volume, hippocampal volume, or mean cortical thickness and when assessed in respect to additional cognitive outcome measures, including verbal fluency, processing speed, and global cognition. Importantly, our results show that this conclusion holds when tested across two separate samples of cognitively healthy older adults.

The larger and more robust effects of verbal intelligence on cognition reported here and elsewhere could be explained by 2 key factors. Firstly, verbal intelligence may be a closer reflection of the *quality*, benefit, or outcomes of educational attainment [112] than years of education, which simply reflects the *quantity* of educational attainment. Quality of education can differ greatly among individuals with the same quantity of education due to various socioeconomic and systemic factors [113], such as class size [114], and also due to individual-level factors such as intrinsic learning motivation and academic self-efficacy [115]. Secondly, measures of verbal intelligence may reflect wider lifetime educational and cognitive experiences as compared to years of education which is generally restricted to early-life formal education [79–82] and typically neglects to consider later-life education which has been positively associated with cognitive function [116, 117]. In this sense, verbal intelligence could be considered a dynamic CR proxy which can change over time [118, 119], as it may increase from young to mid-adulthood before decreasing in older adulthood [120]. In contrast, years of education may be considered a static CR proxy [31]. Despite the widespread use of educational attainment as an individual CR proxy, our results suggest that it should only be used as an individual proxy where verbal intelligence is not available.

### Composite proxies had smaller and less robust effects on cognition than verbal intelligence

We found significant positive independent effects of 16 different composite proxies on cognition across both datasets. Three of these composites had significant effects on cognition in at least two-thirds of the brain-cognition models assessed: occupational complexity and verbal intelligence (86.67% of models); education and verbal intelligence (80% of models); and education, occupational complexity, and verbal intelligence (66.67% of models). This is a novel finding as the most robust composite—occupational complexity and verbal intelligence—has never (to the best of our knowledge) been used previously as a CR proxy, likely due to the predominant use of education both as an individual proxy and in composites. The next most robust composite of education and verbal intelligence has been widely used [42, 43, 45, 46, 59, 77, 111] and our results support a previous positive association between this composite and episodic memory, controlling for GM volume [59]. A speculative explanation for the greater robustness of occupational complexity and verbal intelligence as a composite may be that occupational complexity and verbal intelligence are less strongly correlated with each other than educational attainment and verbal intelligence (see Fig. 2).

While composite proxies purportedly provide advantages over individual proxies, our results show that their independent effects on cognition are less robust (i.e. less frequently observed across brain-cognition models) and smaller in magnitude than those found for verbal intelligence alone. This may be explained by the large individual effects of verbal intelligence on cognition and its strong correlation with other proxies (see Fig. 2) considering that all composite proxies with replicated effects contained verbal intelligence, except for the composite with the least robust effects, occupational complexity and physical activity. While adding another proxy to verbal intelligence to form a composite should have an additive effect, this could also add noise to an already strong proxy measure as well as shared variance in situations where the proxies are correlated. Consequently, the overall effect of the composite may then be smaller than verbal intelligence alone. Alternative methods to creating composites, such as principal component analysis, could potentially mitigate this issue but may not be theoretically appropriate [35], and incorporating this method within the analysis framework used here would have significantly increased the complexity of the analysis. Of all composites considered here, our results especially support the use of education and verbal intelligence as well as occupational complexity and verbal intelligence as composite proxies where multiple proxies are available. However, using composites may lead to more type II errors than using verbal intelligence alone, given the more robust and larger effects of verbal intelligence. As such, our results suggest that researchers should use, or at least repeat analyses using, verbal intelligence alone, in cross-sectional studies of cognitively healthy older adults.

### Occupational complexity, leisure activities, and physical activity did not show robust effects on cognition

We did not find any evidence for robust independent effects of 3 individual proxies on cognition across both datasets. Occupational complexity was not positively associated with any domain of cognitive function, adjusting for GM volume, hippocampal volume, or mean cortical thickness. This suggests that the small positive associations between this proxy and cognition, as reported in a meta-analysis [50], may not be independent of these measures of brain structure. Unlike the detailed nature of the occupational complexity measure used here, occupational complexity has been typically measured using government classification codes that are effectively a socioeconomic classification of occupations (e.g. the UK’s Office Of Population Statistic classification as in Staff et al. [121]). As such, previously reported effects for occupational complexity may have in fact reflected the effect of socioeconomic status, which can support cognitive health via greater access to resources and healthcare, among many other mechanisms [35]. While Chapko et al. [53] concluded that the evidence for this proxy in complete CR models using cognitively healthy samples was inconclusive, our results, do not support the use of occupational complexity as a proxy measure of CR in cross-sectional studies of cognitively healthy older adults.

As with occupational complexity, we did not find robust evidence to support the use of leisure activities as an individual CR proxy. Although it has been associated with a reduced risk of dementia and AD ([122], but cf. [123]), few studies have rigorously tested this proxy in a complete CR model. One study found a moderation effect for midlife leisure activities, but in line with our findings, they did not find evidence of either a moderation or independent effect for later-life leisure activities [124]. Future research is warranted to clarify which specific leisure activities should be included in measures for this proxy given that only a few activities have been associated with cognition in mid-/old-age samples, albeit without adjusting for brain structure [116, 125]. However, our results do not support the use of later-life leisure activities as a proxy measure of CR in cross-sectional studies of cognitively healthy older adults.

Finally, our results do not support the use of physical activity as an individual CR proxy. While this proxy has been previously associated with cognitive function in older adults without controlling for brain structure [106, 126], our results show that these associations are not independent of GM volume, hippocampal volume, or mean cortical thickness. This supports previous findings of non-significant associations from the few complete CR models assessing this proxy adjusting for brain structure using GM volume and hippocampal atrophy [56, 100]. The disparity in the observed associations when brain structure is accounted for could be because the protective effects of exercise may be exerted via improved brain maintenance, i.e. the relative preservation of brain structural health [8, 127], rather than improved CR [128]. This is supported by the finding that the protective effects of exercise on cognition were mediated by increases in prefrontal cortex volume [129] and also by associations of greater physical activity with lower brain-predicted age difference scores [130], which reflects better brain maintenance [131], and greater cortical thickness [132] and regional GM volumes [133, 134]. Setting aside a possible contribution of physical activity to brain maintenance, our results suggest that it does not contribute to greater CR and therefore do not support the use of physical activity as a proxy measure of CR in cross-sectional studies of cognitively healthy older adults.

### Lack of evidence for moderation effects of CR proxies

Robust moderation effects of CR proxies on the association between brain structure—as measured by GM volume, hippocampal volume, or mean cortical thickness—and cognition were not identified here. This lack of evidence is in line with previously reported non-significant moderation effects on the relationship between episodic memory and GM volume [59] and right hippocampal volume [102] but conflicts with previous evidence of significant moderation effects reported for CR proxies in similar brain-cognition models [46, 124, 135]. However, the evidence for moderation is largely inconsistent as highlighted by the finding of moderation effects reported on 1 measure, but not on 2 other measures, of episodic memory within the same study [135] and even findings of a positive moderation effect, which contradicts the CR hypothesis, on the relationship between left hippocampal volume and episodic memory [102]. It is likely that our non-significant effects highlight the general difficulties in detecting CR moderation effects.

The ability to detect a moderation effect here may have been impaired because the participants were cognitively and neurologically healthy and therefore had a relatively restricted range of cognitive function and brain atrophy in comparison to cognitively and/or neurologically impaired individuals. The relatively restricted range of the predictor variable of brain structure restricts the range of the interaction term [136] which can substantially reduce statistical power to detect a moderation effect [137]. This is exacerbated by the fact that neuroimaging variables explain a relatively small amount (20%) of variance in healthy older adults’ cognition [2], which effectively constrains the size of the moderation effect [62]. While the present study was designed using pre-existing data from two cognitively and neurologically healthy cohorts, an experimental approach where individuals with extremely low or high scores on measures of cognitive reserve and brain structure are oversampled may be better able to detect the existence of a moderation effect for these proxies [136].

### Promising evidence for cognitively stimulating activities but not social engagement as proxies but replication required

We were unable to assess the reproducibility of the effects of cognitively stimulating activities and social engagement on cognition across datasets as we only had sufficient data in TILDA for these proxies. Within TILDA, cognitively stimulating activities was highly robust as it had positive independent effects on cognition in all brain-cognition models, and had the largest average independent effect on cognition after verbal intelligence. This finding supports associations between this proxy and neuropathological ‘residual’ measures of CR [54, 55] and suggests that previously reported consistent positive associations [49, 50] can be observed with several cognition domains when controlling for brain structure, as measured by GM volume, hippocampal volume, and mean cortical thickness. Social engagement was less robust as it had positive independent effects on cognition in only 40% of brain-cognition models and had the second smallest average independent effect on cognition of all individual proxies. This inconsistent evidence emphasizes a need for further study of social engagement in complete CR models. While mixed evidence of moderation effects has been reported to date for this proxy controlling for neuropathology [58, 138], this is the first attempt to assess it in a complete CR model including neuroimaging variables. As our focus was on replication across datasets rather than single dataset findings requiring correction for multiple comparisons and because this proxy was only available in a single dataset, these findings remain speculative until they can be replicated. With this in mind, while we cannot make definitive conclusions, we can tentatively suggest that cognitively stimulating activities may be a reasonable choice of CR proxy where verbal intelligence is not available and that social engagement should not be used as an individual proxy.

### Limitations

The present study provides data-driven evidence supporting the use of specific proxies to measure CR in cross-sectional studies of cognitively healthy older adults. Nonetheless, there are some limitations which, if addressed in future research, could further strengthen these recommendations and provide additional insights. The main limitation of the present results is that they are cross-sectional. As such, we cannot make solid inferences about the casual direction of the relationships between the robust proxies and cognitive function. Similarly, while CR is supposed to protect against cognitive decline, our analysis only provides information about its association with individual differences in cognitive function, not decline. Future analyses after further waves of data collection will be necessary to assess whether the effects of these proxies are consistent when assessed in the context of cognitive decline.

Another limitation is that the CR models used here were limited to three brain structure variables: GM volume, hippocampal volume, and mean cortical thickness. Aside from hippocampal volume, the CR models did not contain regional measures such as parietotemporal cortical thickness or measures of WM microstructural integrity, WM hyperintensity volume, or AD-related neuropathology. As CR proxies have been previously reported to moderate the relationship between these measures and cognition [43, 82, 139–142], future studies could assess proxies in complete CR models containing these brain structure variables to extend the conclusions made here to a wider spectrum of brain-cognition relationships. Furthermore, there were differences in the relationship between age and cognition across both datasets. Age was negatively associated with cognition in 100% of brain-cognition models in TILDA, but only in 40% of models in CR/RANN. Tentative explanations for these differences may have been the larger sample size and older age of the TILDA brain-cognition models. Finally, some CR proxies, namely leisure activities and physical activity were measured differently in both datasets. Differences in these measures or in the specific activities included in each measure may have contributed to differing effects across both datasets. This may be particularly pertinent for leisure activities as its relationship with cognitive function can vary based on the specific leisure activities assessed [116]. However, this variability across the two datasets reflects the typical variability in the measurement of CR with proxies.

## Conclusions

Despite the discussed limitations, the present findings are informative for researchers using proxies as measures of CR. We built on previous meta-analyses and systematic reviews of CR proxies by assessing a wider set of standard proxies, including their composites, and evaluating their effects across complete and theoretically consistent models of CR and in multiple brain-cognition relationships. Our analysis framework enabled the comparison of the robustness and magnitude of effects. Furthermore, the reported findings are stringent, robust, and replicable, as they were only considered statistically significant if they were replicated in a robust regression and across two datasets.

The present study is the first systematic investigation of the validity of different proxies, and their composites, in complete CR models. Verbal intelligence was associated with better cognitive function in all variables assessed, controlling for mean cortical thickness, GM volume, and hippocampal volume. The independent effects on cognition of education and composite proxies, including verbal intelligence and occupational complexity as well as verbal intelligence and education, were smaller and less robust. Our results suggest that, in cross-sectional studies of cognitively healthy older adults, verbal intelligence should be used as a CR proxy, over other proxies including education, occupational complexity, leisure activities, exercise, and composites including all possible combinations of these proxies. While no robust moderation effects of CR proxies on the association between brain structure—as measured by GM volume, hippocampal volume, or mean cortical thickness—and cognition were found here, this may be due to the considerable statistical difficulties in detecting such effects in normal healthy ageing samples. In sum, the finding of robust independent effects across all brain-cognitive domains assessed provides strong evidence for the use of verbal intelligence as a CR proxy.

## Supplementary Information

**Additional file 1.** Significant independent effects across both datasets.

**Additional file 2.** All significant independent effects in TILDA.

**Additional file 3.** All significant independent effects in CR/RANN.

**Additional file 4 Table S1** Negative moderation effects of cognitive reserve proxies within TILDA. **Table S2** Positive moderation effects of cognitive reserve proxies within both datasets. **Figure S1:** Association between proxies and cognition, adjusting for brain structure, age, and sex*.*

## Data Availability

The TILDA dataset analysed in this study is available from TILDA upon reasonable request. The procedures to gain access to TILDA data are specified at https://tilda.tcd.ie/data/accessing-data/.

## Abbreviations

AD: Alzheimer’s disease
AMNART: American National Adult Reading Test
CR: Cognitive reserve
CR/RANN: The Cognitive Reserve/Reference Ability Neural Network Study
CTT: Colour Trails Test
DOT: Dictionary of Occupational Titles
FOV: Field of view
GM: Grey matter
IPAQ-SF: International Physical Activity Questionnaire-Short Form
MCI: Mild cognitive impairment
MRI: Magnetic resonance imaging
MPRAGE: Magnetization-prepared rapid acquisition with gradient echo
NART: National Adult Reading Test
ROI: Region of interest
TILDA: The Irish Longitudinal Study on Ageing
TMT: Trail Making Test
TR/TE: Repetition time/echo time
WM: White matter
