# Supplementary material for: Verbal intelligence is a more robust cross-sectional measure of cognitive reserve than level of education in healthy older adults
Source: Alzheimers Res Ther. 2021 Jul 12;13:128. doi: 10.1186/s13195-021-00870-z (PMC8276413; doi:10.1186/s13195-021-00870-z)
Supplement: Supplementary file 4 — Additional file 4 Table S1 Negative moderation effects of cognitive reserve proxies within TILDA. Table S2 Positive moderation effects of cognitive reserve proxies within both datasets. Figure S1: Association between proxies and cognition, adjusting for brain structure, age, and sex. [file 13195_2021_870_MOESM4_ESM.docx]

**Table S1** Negative moderation effects of cognitive reserve proxies within TILDA

| **Brain Structure** | **Cognition** | **Cognitive Reserve Proxy** | **n** | ***ΔR*^2^** | ***β*** |
| --- | --- | --- | --- | --- | --- |
| Cx Thickness | Epi Mem | Occu + CogStim + Ex | 279 | .034 | -.340** |
| Cx Thickness | Epi Mem | Occu + CogStim + Leisure + Ex | 279 | .032 | -.335** |
| Cx Thickness | Epi Mem | Occu + Leisure + Ex | 279 | .030 | -.310** |
| Cx Thickness | Epi Mem | CogStim + Ex | 279 | .029 | -.286** |
| Cx Thickness | Epi Mem | Leisure + Ex | 279 | .026 | -.245** |
| Cx Thickness | Epi Mem | CogStim + Leisure + Ex | 279 | .025 | -.258** |
| Cx Thickness | Epi Mem | Occu + Ex | 279 | .022 | -.242** |
| Cx Thickness | Epi Mem | Edu + Occu + CogStim + Ex | 279 | .021 | -.270** |
| Cx Thickness | Epi Mem | Occu + CogStim | 279 | .021 | -.214* |
| Cx Thickness | Epi Mem | Edu + Occu + CogStim + Leisure + Ex | 279 | .020 | -.265* |
| Cx Thickness | Epi Mem | Edu + Occu + Leisure + Ex | 279 | .019 | -.255* |
| Cx Thickness | Epi Mem | Ex | 279 | .018 | -.200* |
| Cx Thickness | Epi Mem | Occu + CogStim + Leisure | 279 | .018 | -.204* |
| Cx Thickness | Epi Mem | Occu + Social + CogStim + Leisure + Ex | 279 | .015 | -.233* |
| GM Volume | Epi Mem | CogStim + Ex | 313 | .015 | -.176* |
| Cx Thickness | Epi Mem | Edu + Occu + Ex | 279 | .015 | -.205* |
| Cx Thickness | Glob Cog | Edu + Leisure + Ex | 277 | .015 | -.198* |
| Cx Thickness | Glob Cog | CogStim + Leisure + Ex | 277 | .014 | -.185* |
| Cx Thickness | Glob Cog | Leisure + Ex | 277 | .014 | -.175* |
| Cx Thickness | Epi Mem | Occu + Social + CogStim + Ex | 279 | .014 | -.227* |
| Cx Thickness | Epi Mem | Occu + Verbal IQ + CogStim + Leisure + Ex | 279 | .013 | -.223* |
| Cx Thickness | Glob Cog | Edu | 277 | .013 | -.113* |
| Cx Thickness | Glob Cog | Edu + CogStim + Leisure + Ex | 277 | .013 | -.187* |
| Cx Thickness | Epi Mem | Occu + Verbal IQ + CogStim + Ex | 279 | .012 | -.212* |
| Cx Thickness | Glob Cog | Edu + Occu + CogStim + Leisure + Ex | 277 | .012 | -.195* |
| GM Volume | Exec Func | Edu + CogStim + Ex | 311 | .011 | -.179* |
| Cx Thickness | Glob Cog | Edu + Leisure | 277 | .011 | -.131* |
| Cx Thickness | Glob Cog | Leisure | 277 | .011 | -.103* |
| Cx Thickness | Glob Cog | Edu + Occu + Leisure + Ex | 277 | .011 | -.189* |
| Cx Thickness | Glob Cog | Occu + CogStim + Leisure + Ex | 277 | .011 | -.191* |
| GM Volume | Glob Cog | Edu + CogStim + Ex | 311 | .010 | -.161* |

*Note: * = p < .05, ** = p < .01 Cx Thickness = Mean Cortical Thickness, GM Volume = Grey Matter Volume, Epi Mem = Episodic Memory, Glob Cog = Global Cognition, Exec Func = Executive Function, Occu = Occupational Complexity, CogStim = Cognitively Stimulating Activities, Ex = Exercise, Leisure = Leisure Activities, Edu = Educational Attainment, Social = Social Engagement, Verbal IQ = Verbal Intelligence.*

**Table S2** Positive moderation effects of cognitive reserve proxies within both datasets

| **Dataset** | **Brain Structure** | **Cognition** | **Cognitive Reserve Proxy** | **n** | ***ΔR*^2^** | ***β*** |
| --- | --- | --- | --- | --- | --- | --- |
| TILDA | HC Volume | Verb Flu | Occu + Ex | 313 | .019 | .227* |
|  | GM Volume | Proc Speed | Occu | 313 | .018 | .148* |
|  | GM Volume | Verb Flu | Occu + Ex | 313 | .017 | .214* |
|  | Cx Thickness | Verb Flu | Occu + Social + Ex | 279 | .015 | .235* |
|  | HC Volume | Verb Flu | Occu + Social + Ex | 313 | .015 | .243* |
|  | HC Volume | Verb Flu | Ex | 313 | .014 | .192* |
|  | GM Vol | Verb Flu | Occu + Social + Ex | 313 | .014 | .231* |
|  | HC Volume | Verb Flu | Social + Ex | 313 | .014 | .208* |
| CR/RANN | HC Volume | Glob Cog | Occu + Verbal IQ | 234 | .030 | .215** |
|  | HC Volume | Epi Mem | Occu + Ex | 234 | .026 | .232* |
|  | HC Volume | Glob Cog | Occu + Verbal IQ + Ex | 234 | .026 | .266** |
|  | HC Volume | Glob Cog | Occu | 234 | .025 | .155* |
|  | HC Volume | Epi Mem | Occu + Verbal IQ + Ex | 234 | .025 | .263* |
|  | HC Volume | Epi Mem | Occu | 234 | .024 | .151* |
|  | HC Volume | Exec Func | Occu + Verbal IQ | 234 | .018 | .168* |

*Note: * = p < .05, ** = p < .01. HC Volume = Hippocampal Volume, GM Volume = Grey Matter Volume, Cx Thickness = Mean Cortical Thickness, Verb Flu = Verbal Fluency, Proc Speed = Processing Speed, Glob Cog = Global Cognition, Epi Mem = Episodic Memory, Exec Func = Executive Function, Occu = Occupational Complexity, Ex = Exercise, Social = Social Engagement, Verbal IQ = Verbal Intelligence.*

|  | **TILDA** | **CR/RANN** |
| --- | --- | --- |
| Glob Cog ~ Intell \| Hipp Vol,  Age, Sex  Mean Δ*R^2^* = .168 | 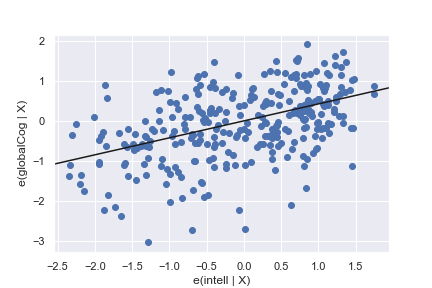 | 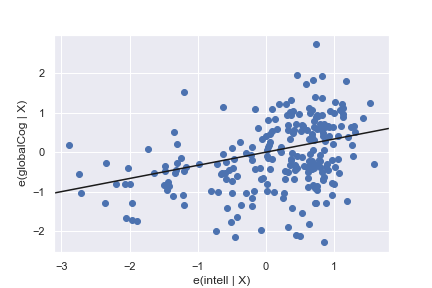 |
| Glob Cog ~ Intell \| GM Vol,  Age, Sex  Mean Δ*R^2^* = .159 | 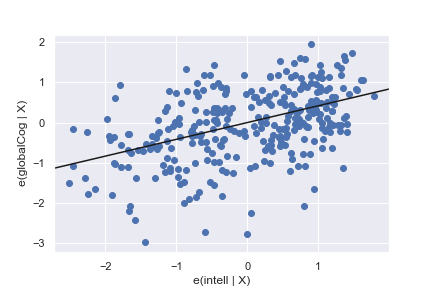 | 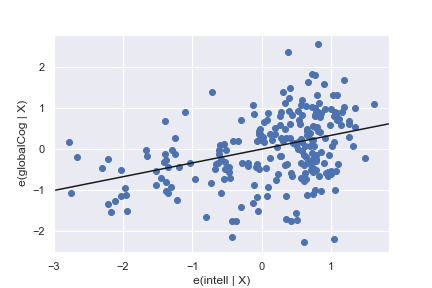 |
| Glob Cog ~ Intell \| Cx Th,  Age, Sex  Mean Δ*R^2^* = .147 | 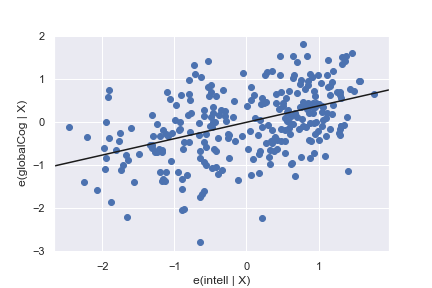 | 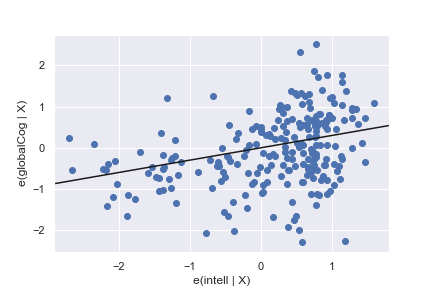 |
| Exec Func ~ Intell  \| Hipp Vol,  Age, Sex  Mean Δ*R^2^* = .146 | 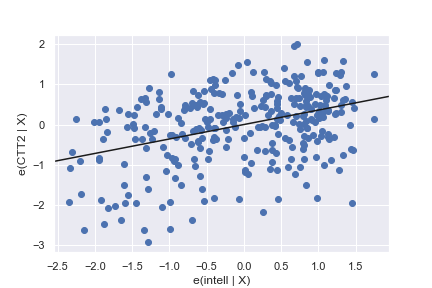 | 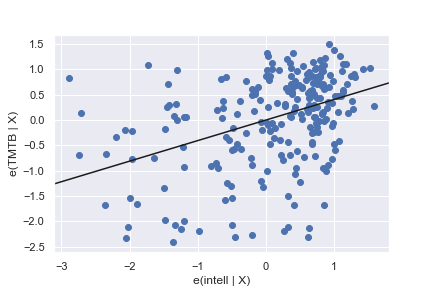 |
| Exec Func ~ Intell  \| GM Vol,  Age, Sex  Mean Δ*R^2^* = .142 | 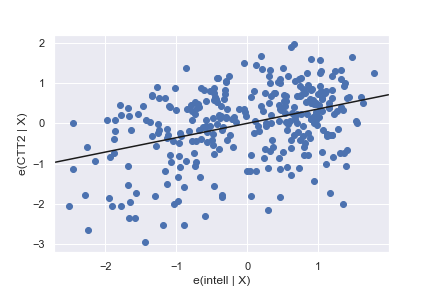 | 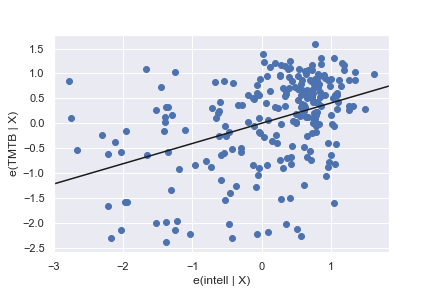 |
|  | **TILDA** | **CR/RANN** |
| Glob Cog ~ Edu_Intell \| Hipp Vol,  Age, Sex  Mean Δ*R^2^* = .136 | 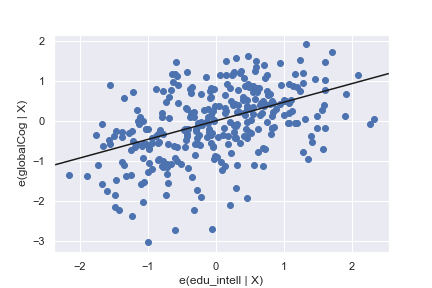 | 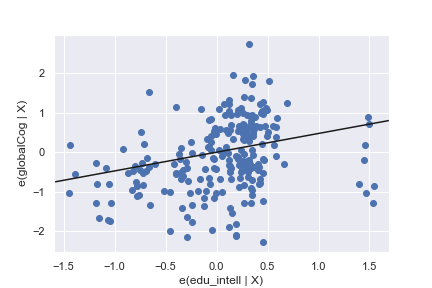 |
| Glob Cog ~ Edu_Intell \| GM Vol,  Age, Sex  Mean Δ*R^2^* = .127 | 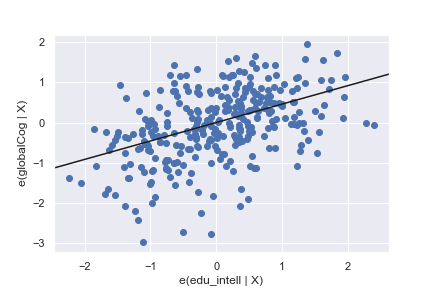 | 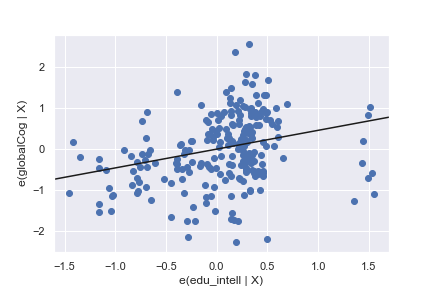 |
| Exec Func ~ Edu_Intell \| Hipp Vol,  Age, Sex  Mean Δ*R^2^* = .123 | 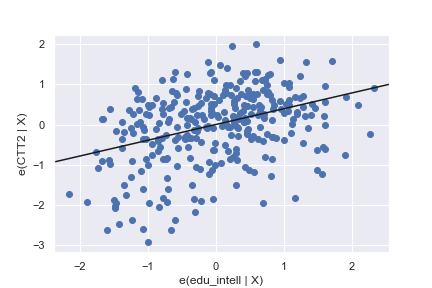 | 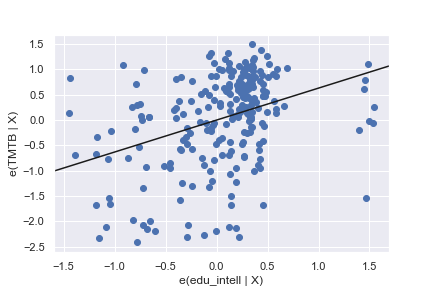 |
| Glob Cog ~ Edu_Intell \| Cx Th,  Age, Sex  Mean Δ*R^2^* = .121 | 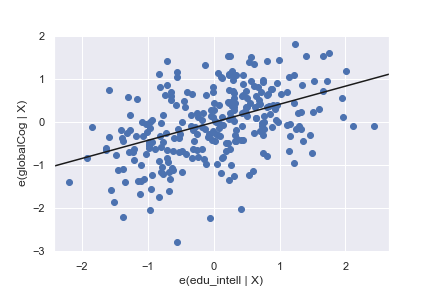 | 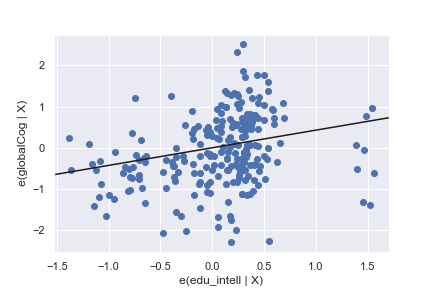 |
| Exec Func ~ Intell \| Cx Th,  Age, Sex  Mean Δ*R^2^* = .120 | 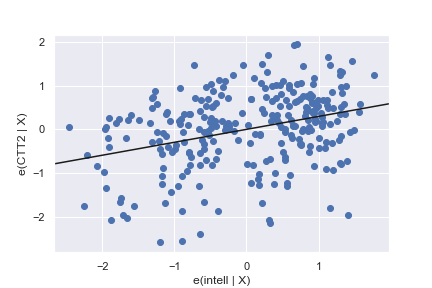 | 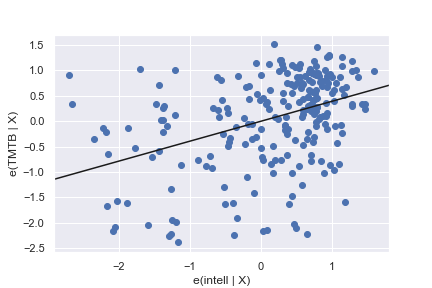 |

**Figure S1:** Association between proxies and cognition, adjusting for brain structure, age, and sex*. Plots are shown for the 10 largest mean R^2^ change across both datasets for proxies with significant effects. Glob Cog = Global Cognition, Exec Func = Executive Function, Intell = Premorbid Intelligence, Edu_Intell = Composite of Education and Premorbid Intelligence, Hipp Vol = Hippocampal Volume, GM Vol = Grey Matter Volume, Cx Th = Mean Cortical Thickness*
